# Epidemiological study of vehicle drowning in Iran: injury and mortality analysis

**Published:** 2024-10

**Authors:** Seyyed Ahmad Mohammadi Kia, Yahya Saleh Tabari, Mehdi Bagheri, Fatemeh Rajabi

**Affiliations:** ^*a*^ Department of Health in Disasters and Emergencies, Shahid Sadoughi University of Medical Sciences, Yazd, Iran.; ^*b*^ Emergency Medical Services, Mazandaran University of Medical Sciences, Sari, Iran.; ^*c*^ Babolsar Health Network, Mazandaran University of Medical Sciences, Sari, Iran.; ^*d*^ Babol University of Medical Sciences, Babol, Iran.

## Abstract

**Background:**

Vehicle drowning represents a significant public health issue in Iran, particularly in regions with extensive canal networks. This study aims to analyze the epidemiological patterns of vehicle drowning, examining factors such as age, gender, geographical distribution, and temporal trends. The purpose is to inform the development of targeted prevention strategies and policy interventions to reduce the incidence and mortality associated with vehicle drowning.

**Methods:**

This study utilizes a longitudinal analysis of vehicle drowning incidents in Iran from various data sources, including the National Death Registration System, the National Forensic Medicine System, hospital-based samples, and Iran’s Demographic and Health Survey from 1990 to 2019. Drowning data from the Legal Medicine Organization of Iran for the years 2005 to 2017 were also included. Statistical analyses involved descriptive statistics, linear mixed-effect models, generalized estimating equations, survival analysis, structural equation modeling, partial least squares, orthogonal PLS, and ARIMA for time series forecasting.

**Results:**

The study found significant demographic variations in vehicle drowning incidents, with higher incidence rates among males (2.6 per 100,000) compared to females (0.4 per 100,000). The age group 15-24 years had the highest incidence rate (3.0 per 100,000). Geographically, certain regions, particularly the northeastern and northwestern margins of Tehran, exhibited clustering of incidents. Seasonal trends indicated that over 91% of drowning deaths occurred between April and September, peaking in July and August.

**Conclusions:**

Vehicle drowning is a critical public health challenge in Iran, necessitating a multifaceted approach encompassing engineering interventions, educational campaigns, and policy changes. Effective measures such as roadway lighting, traffic calming, multiway stop signs, roundabouts, and targeted educational programs can significantly reduce the incidence of vehicle drowning. Further research is essential to develop evidence-based prevention strategies and improve road safety comprehensively.

**Keywords:**

Vehicle drowning, Epidemiology, Prevention strategies, Mortality, geographical distribution Iran

